# Interest in treatment with injectable diacetylmorphine among people who use opioids in Baltimore City, Maryland (USA)

**DOI:** 10.1080/07853890.2023.2196435

**Published:** 2023-04-20

**Authors:** Sean T. Allen, Kristin E. Schneider, Saba Rouhani, Rebecca Hamilton White, Miles Morris, Jill Owczarzak, Susan G. Sherman

**Affiliations:** Department of Health, Behavior and Society at the Johns Hopkins Bloomberg School of Public Health, Baltimore, MD, USA

**Keywords:** Opioid use disorder, medications for opioid use disorder, treatment with injectable diacetylmorphine

## Abstract

**Introduction:**

Treatment for opioid use disorder (OUD) with diacetylmorphine is an evidence-based form of drug treatment, but it is not available in the United States (US). Better understanding acceptability of treatment with injectable diacetylmorphine among people who use opioids (PWUO) in the US may expedite future initiatives designed to engage persons in this form of treatment should it become available. The purpose of this research is to examine factors associated with interest in treatment with injectable diacetylmorphine among a sample of PWUO in the US.

**Methods:**

Data are from a cross-sectional study of PWUO in Baltimore City, Maryland. Participants were given a brief description of treatment with injectable diacetylmorphine and then asked to rate their level of interest. We used Poisson regression with robust variance to assess factors associated with interest in treatment with injectable diacetylmorphine.

**Results:**

The average age of participants was 48 years, 41% were women, and most (76%) identified as non-Hispanic, Black. The most commonly used substances were non-injection heroin (76%), opioid pain relievers (73%), and non-injection crack/cocaine (73%). Two-thirds of participants (68%) indicated interest in treatment with injectable diacetylmorphine. Factors significantly associated with interest in injectable diacetylmorphine treatment included: having at least a high school education (adjusted prevalence ratio [aPR]: 1.23; 95% confidence interval [CI]: 1.04–1.45), not having health insurance (aPR: 1.23; 95% CI: 1.06–1.44), having ever overdosed (aPR: 1.20; 95% CI: 1.01–1.42), and past utilization of medications for opioid use disorder (aPR: 1.22; 95% CI: 1.01–1.47). Recent non-injection cocaine use was inversely associated with interest in treatment with injectable diacetylmorphine (aPR 0.80; 95% CI: 0.68–0.94).

**Conclusion:**

The majority of participants reported interest in treatment with injectable diacetylmorphine. Given worsening trends in the addiction and overdose crisis in the US, treatment with injectable diacetylmorphine should be considered as another evidence-based option for treating OUD.

**KEY MESSAGES**
- Interest in treatment with injectable diacetylmorphine was high among a sample of people who use opioids in the United States.
- Factors associated with increased interest in treatment with injectable diacetylmorphine included having at least a high school education, having ever overdosed, and not having health insurance.
- Past utilization of medications for opioid use disorder was associated with interest in treatment with injectable diacetylmorphine.

## Introduction

In 2020, approximately 2.5 million people aged 12 or older in the United States (US) had an opioid use disorder (OUD) in the past year [1]. The prevalence of OUD in the US is alarming given worsening trends in overdose mortality [2–5]. Provisional data suggest in the year ending in April 2021 there were more than 100,000 overdose fatalities in the US with most involving opioids [3]. Overdose mortality data reflects only one measure of the harms associated with OUD. Opioid-involved non-fatal overdoses may also result in significant health consequences, including brain injury, respiratory depression, cardiac and renal problems, and acute traumatic injuries [6–8]. In addition, opioid misuse has been associated with engagement in high-risk behaviors for bloodborne infectious disease acquisition (e.g. syringe sharing) and injection drug use-associated HIV outbreaks among people who inject drugs [9–13]. Taken together, there are sustained needs for increased access to evidence-based substance use treatment options in the US.

Medications for opioid use disorder (MOUD), such as methadone and buprenorphine, are considered the gold standard for treating OUD as their utilization has been shown to reduce opioid use, overdose mortality, healthcare costs, and high-risk behaviors associated with infectious disease transmission [14–18]. Despite their efficacy, most persons with an OUD in the US do not utilize MOUD; in 2020, only 11% of persons aged 12 or older with an OUD received MOUD [1]. This low level of MOUD utilization likely reflects a combination of factors, including stigma, lack of MOUD prescribers, cost, and high thresholds for service access [1,19–21]. Further, MOUD treatment systems in the US are often punitive, not patient centered, and require frequent visits [22–25]. Additionally, research has shown that not all people who use opioids (PWUO) respond favorably to MOUD as persons may be unable to reach therapeutic MOUD dosages or tolerate side effects and discontinue medication use [26–28]. A recent systematic review, for instance, found that retention in MOUD among persons with opioid dependence varied considerably, from: 19%–94% at 3-months, 46%–92% at 4-months, 3%–88% at 6-months, and 37%–91% at 12-months [29].

For persons with severe OUD who are unable to maintain utilization of methadone or buprenorphine, treatment with diacetylmorphine may be a viable alternative [30–36]. This form of MOUD, sometimes referred to as ‘heroin assisted treatment’, involves providing persons with an OUD pharmaceutical grade diacetylmorphine as an oral tablet or injectable liquid [30–36]. Treatment with diacetylmorphine has been studied for many years internationally. It was first introduced in Switzerland in 1994 and has since been made available in eight other countries [37,38]. Studies have demonstrated a myriad of benefits associated with diacetylmorphine treatment utilization, including increased treatment retention and reductions in: illicit opioid use, criminal activity, and overdose fatalities [30–37,39,40]. Unfortunately, diacetylmorphine-based treatment is not available in the US due to a range of policy and ideological barriers, including stigma, regulations that obstruct prescribing controlled substances, and cost [41].

Given the scale of the addiction and overdose crisis in the US, ensuring persons have access to an array of MOUD formulations is vital to ensuring public health. Diacetylmorphine-based treatment may be particularly efficacious for persons who were not able to maintain utilization of other MOUD formulations, including methadone and buprenorphine [40,42,43]. For example, diacetylmorphine has shown more effective than methadone for persons with chronic treatment-refractory heroin dependence [44–46]. For instance, a 2009 randomized controlled trial among persons who injected heroin in Canada found that rates of illicit-drug use were significantly lower among persons who received injectable diacetylmorphine than persons receiving oral methadone [46]. Diacetylmorphine in combination with other MOUD formulations has also shown efficacious. For example, a study conducted in Spain found that providing persons with opioid dependence injectable diacetylmorphine and oral methadone was associated with greater reductions in HIV risk behaviors, illicit heroin use, and days involved in crime than methadone alone [47]. In the US, the implementation of diacetylmorphine-based treatment may also yield added benefits in terms of overdose reduction as this form of pharmacotherapy may lead to reductions in the purchase of illicit drugs that may be adulterated with other substances, such as fentanyl [41]. Further, if the provision of diacetylmorphine-based treatment in the US mirrored that of other countries, persons would be required to visit medical facilities where they may access other support services, such as housing assistance, harm reduction resources, and medical services [48]. By offering diacetylmorphine- based treatment at facilities that aim to address the totality of unmet needs *via* the co-location of comprehensive wrap-around services, patients may glean additional benefits as persons with drug dependence often have high rates of unstable housing, food insecurity, and comorbidities.

Similar to other pharmacologic approaches to OUD [49], diacetylmorphine-based treatment is not without potential complications and side effects. Persons receiving intramuscular injections of diacetylmorphine for OUD in Switzerland, for instance, reported indurations, pain at the injection site, and muscle pain [30]. Skin reactions, dizziness, and respiratory difficulties have also been documented [38]. Notably, evidence suggests diacetylmorphine-based treatment has greater risks than oral methadone, including respiratory depression and seizures [48]. These risks, however, should be considered in combination with potential requirements related to the administration of diacetylmorphine-based treatment. For example, in some countries, persons are required to visit a healthcare facility to utilize diacetylmorphine-based treatment [43]. Given that medical professionals would be onsite, risks for serious adverse effects are minimized [48]. Importantly, legal and regulated sources of diacetylmorphine would carry less risks for overdose and other adverse side effects than unregulated and illicit drug supplies [43].

Developing and implementing evidence-based forms of substance use disorder treatment represents a high-priority realm of scientific inquiry given the magnitude of the addiction and overdose crisis. International research documents the public health utility of treatment with diacetylmorphine among persons with OUD; however, as of 2022, this form of treatment is not available in the US. Better understanding acceptability for treatment with injectable diacetylmorphine among PWUO in the US may expedite future initiatives designed to engage persons in this form of treatment should it become available. The purpose of this research is to describe interest in treatment with injectable diacetylmorphine among a sample of PWUO in Baltimore City, Maryland (USA). Second, we examined factors associated with interest in treatment with injectable diacetylmorphine among our sample of PWUO in Baltimore City, Maryland. We hypothesized that PWUO would express substantial interest in treatment with injectable diacetylmorphine and that interest would be associated with prior experiences with MOUD-based treatment.

## Methods

### Study overview

Data are from the ‘Peer harm Reduction of Maryland Outreach Tiered Evaluation’ (PROMOTE) study, a cross-sectional study of people who used non-prescription opioids in Baltimore City, Maryland (US) and adjacent Anne Arundel County [50,51]. The overall goal of the PROMOTE study was to evaluate the impact of a direct peer outreach program on access to harm reduction services among PWUO. Detailed methodological procedures, including survey instrument development, are described elsewhere, and we present a brief description below [50–52]. The Baltimore City components of the PROMOTE study were comprised of two cross-sectional waves with the first wave spanning July 2018 to October 2018. Wave 2 launched April 2019 and ended in July 2019. This analysis was limited to the second wave of data collection in Baltimore City given that it included the outcome measure for the present analysis.

Recruitment occurred throughout Baltimore City in areas with concentrated drug use activity. Similar to another study, we identified these areas *via* geospatial analyses of publicly available arrest data; specifically, heat maps were created to understand the geospatial distribution of drug-related arrests in Baltimore City [53]. Within areas of concentrated drug-related arrests, we extracted the time signatures of arrest events to develop a sampling frame. Staff then parked a study van in the identified locations at times with peak arrest activity and approached individuals about potential study participation. Eligibility criteria for study participation in Baltimore City were the following: (1) at least 18 years of age; and (2) used any opioid (i.e. heroin, prescription opioid pain analgesics, fentanyl/fentanyl analogues) not as prescribed by a healthcare provider in the past month. After providing written informed consent to enroll, participants completed a 30-minute Audio Computer-Assisted Self-Interviewing (ACASI) survey. The survey included questions pertaining to sociodemographics, structural vulnerabilities, substance use behaviors, and drug treatment. Respondents received a pre-paid $25 VISA gift card for study participation. All study procedures and instruments were reviewed and approved by the Johns Hopkins Bloomberg School of Public Health Institutional Review Board.

### Measures

#### Primary outcome

To assess interest in treatment with injectable diacetylmorphine, participants were first given a brief description of treatment with injectable diacetylmorphine, ‘Some countries legally prescribe injectable heroin as a drug treatment option’. They were then asked, ‘How interested would you be in receiving medical-grade heroin from a doctor or nurse?’ Answer options included: not interested at all, somewhat interested, interested, and very interested. Participants were also given options to select ‘don’t know’ and refuse to answer. We dichotomized responses to any interest (somewhat interested, interested, or very interested) versus not at all interested. We chose to dichotomize given that this formulation of treatment may not be medically appropriate for all persons and warrants discussion with healthcare providers. As a result, any expression of interest versus not was considered an important and necessary first step in this line of scientific inquiry. Four persons were excluded as two responded ‘don’t know’ and two refused to answer the question. We only asked about injectable diacetylmorphine as this was an exploratory line of inquiry and we did not want to substantially increase participant burden by asking about other formulations.

#### Sociodemographics

Sociodemographics included basic information about participants, including age (continuous in years), racial and ethnic identity, gender (woman vs. man), relationship status (in a relationship or married vs. single), and sexual minority identity. For their racial and ethnic identity, participants were asked to select all that apply from the following list: White, Black or African American, Hispanic/Latino, Asian/Pacific Islander, Native American/Alaskan Native, or other. Given sample size constraints, we trichotomized these options to non-Hispanic White, non-Hispanic Black/African American, or Hispanic or other racial/ethnic identity. Sexual minority identity was defined as identifying as gay, lesbian, bisexual, or other versus heterosexual or straight.

#### Structural vulnerabilities

We asked about highest educational attainment (high school diploma, GED or more education vs. not graduated high school), homelessness in the past 6 months, arrest in the past year, and if participants currently have health insurance (yes/no). We defined food insecurity as going to bed hungry at least once per week in the past 3 months because there was not enough food. Transactional sex work was defined as selling or trading oral, vaginal, or anal sex for money, drugs, favors, or material assistance in the past 3 months.

#### Substance use

Participants reported how frequently they used different drugs in the past 3 months. Based on this, we created a binary indicator for if a participant had used each substance at least once per month. Tranquilizers or benzodiazepines included Klonopin, Xanax, Ativan, Phinergans, and Valium. Prescription stimulants included substances such as Adderall, Ritalin, Focalin, Concerta, Dexedrine, dextroamphetamine. We also asked about non-injection crack or cocaine, non-injection fentanyl, non-injection fentanyl and cocaine together, and non-injection heroin. Participants further reported if they had injected any drugs at least monthly in the past 3 months. We defined public drug use as use in any of the following locations in the past 3 months: street/park, an abandoned building, a shooting gallery, car/vehicle/bus/metro, stairwell, or a public bathroom. We also asked participants if they had ever overdosed and if they had experienced an overdose in the past 6 months.

#### Drug treatment

Persons reported if they had ever engaged in drug treatment which was defined as a drug treatment or drug detoxification program. In a subsequent question, we asked persons when they were last in a residential rehabilitation program. Answer options were dichotomized to reflect persons who had ever been in a rehabilitation program versus never. We also asked if persons had participated in Narcotics Anonymous or a 12-Step program in the past six months (yes/no). We also asked about MOUD utilization, defined as having received any of the following medications as part of drug treatment: methadone, buprenorphine, and naltrexone. In a follow-up question, we asked persons the recency with which they received these medications as part of their drug treatment. We trichotomized responses based on when participants indicated receiving MOUD: currently receiving MOUD; past receipt of MOUD (ever received MOUD for treatment but not currently); never received MOUD.

#### Statistical analysis

We used Pearson’s chi-square tests and independent sample t-tests for categorical/binary and continuous variables, respectively, to assess associations between variables of interest and interest in treatment with injectable diacetylmorphine. Variables with a p-value <0.10 in the bivariate analyses were considered for inclusion in the multivariable model. We excluded homelessness as it was highly correlated with food insecurity, and food insecurity yielded better model fit statistics (i.e. AIC, BIC). We used Poisson regression with robust variance for the multivariable model. We used this analytical approach as it is valid for estimating prevalence ratios in cases with high prevalence and modest sample size, such as in our study, and may produce less biased estimates than log binomial regression analyses [54]. All statistical analyses were conducted using Stata/SE v15.1 (StataCorp, College Station, TX).

## Results

The average age of our sample was 48 years, women represented 41% of our participants, and most (76%) persons identified as non-Hispanic, Black (Table 1). Structural vulnerabilities were common among our sample: 61% were food insecure, 72% experienced homelessness in the past 6 months, and 17% had been arrested in the past year. However, a small proportion (19%) reported not having health insurance. The most commonly reported substances used at least once per month in the past three months were: non-injection heroin (76%), opioid pain relievers (73%), and non-injection crack/cocaine (73%).

**Table 1.** Interest in treatment with injectable diacetylmorphine among people who use opioids; Baltimore City, Maryland (US), April–July 2019.

	Total	Interest in treatment with diacetylmorphine		
		Interested	Not interested	
	*n* = 287; *n* (%)	*n* = 195, 67.9%; *n* (%)	*n* = 92, 32.1%; *n* (%)	*p*-Value
SOCIODEMOGRAPHICS				
Age, mean (*SD*)	48.0 (10.6)	47.1 (11.0)	49.9 (9.5)	**0.034**
Race				**0.044**
Non-Hispanic, White	37 (13.1)	30 (15.5)	7 (7.8)	
Non-Hispanic, Black	216 (76.3)	139 (72.0)	77 (85.6)	
Hispanic or other	30 (10.6)	24 (12.4)	6 (6.7)	
Woman	117 (41.1)	76 (39.4)	41 (44.6)	0.405
In a relationship/married	82 (28.6)	54 (27.7)	28 (30.4)	0.631
Sexual minority	44 (15.4)	27 (13.9)	17 (18.5)	0.318
STRUCTURAL VULNERABILITIES				
High school diploma/GED or higher education	179 (62.6)	130 (67.0)	49 (53.3)	**0.025**
Homeless, past 6 months	207 (72.1)	147 (75.4)	60 (65.2)	0.073
Arrest, past year	50 (17.4)	36 (18.5)	14 (15.2)	0.499
Food insecurity	175 (61.0)	128 (65.6)	47 (51.1)	**0.018**
Transactional sex work	83 (28.9)	56 (28.7)	27 (29.4)	0.913
No health insurance	53 (18.5)	8 (8.7)	45 (23.2)	**0.003**
SUBSTANCE USE				
Opioid pain relievers^a^	210 (73.2)	141 (72.3)	69 (75.0)	0.631
Tranquilizers or benzodiazepines^a^	127 (44.4)	85 (43.8)	42 (45.7)	0.770
Prescription stimulants^a^	75 (26.2)	47 (24.2)	28 (30.4)	0.265
Prescription gabapentin^a^	85 (29.9)	52 (26.9)	33 (36.3)	0.10
Non-injection crack/cocaine^a^	210 (73.2)	136 (69.7)	74 (80.4)	0.056
Non-injection fentanyl^a^	155 (54.0)	105 (53.9)	50 (54.4)	0.937
Non-injection fentanyl & cocaine together^a^	130 (45.3)	84 (43.1)	46 (50.0)	0.272
Non-injection heroin^a^	218 (76.0)	149 (76.4)	69 (75.0)	0.794
Injection drug use^a^	105 (36.6)	81 (41.5)	24 (26.1)	**0.011**
Use drugs in public location, past 3 months	195 (68.2)	142 (73.2)	53 (57.6)	**0.008**
Ever overdosed (OD)	153 (53.3)	114 (58.5)	39 (42.4)	**0.011**
OD, past 6 months	79 (27.5)	57 (29.2)	22 (23.9)	0.347
DRUG TREATMENT				
Ever drug treatment	226 (78.7)	154 (79.0)	72 (78.3)	0.890
Ever residential rehab program	194 (67.6)	134 (68.7)	60 (65.2)	0.554
Narcotics Anonymous or 12-Step program, past six months	126 (44.1)	83 (42.6)	43 (47.3)	0.457
Medication for Opioid Use Disorder (MOUD) Utilization				**0.001**
Never MOUD	81 (28.3)	52 (26.7)	29 (31.9)	
Past MOUD	124 (43.4)	98 (50.3)	26 (28.6)	
Currently receiving MOUD	81 (28.3)	45 (23.1)	36 (39.6)	

^a^At least monthly.

Bold values signify *p*<.05.

Overall, two-thirds of participants (68%) indicated interest in treatment with injectable diacetylmorphine (25% reported being ‘very interested’, 18% reported being ‘interested’, and 24% reported being ‘somewhat interested’). A significantly (*p*<.05) greater proportion of participants who reported interest in treatment with injectable diacetylmorphine as compared to persons uninterested had at least a high school education (67% vs. 53%) and reported food insecurity (66% vs. 51%). A smaller proportion of persons interested in treatment with injectable diacetylmorphine reported not having health insurance than their counterparts who were not interested (9% vs. 23%). Prevalence of injection drug use was significantly greater among participants interested in treatment with injectable diacetylmorphine versus persons who were not interested (42% vs. 26%). Similarly, a greater proportion of persons interested in treatment with injectable diacetylmorphine reported recently using drugs in a public location (73% vs. 58%). Nearly 80% of our sample reported having ever been in a drug treatment program. Half of participants interested in treatment with injectable diacetylmorphine reported past engagement in MOUD-based treatment compared to only 29% of participants not interested in treatment with injectable diacetylmorphine. While we did not find a difference in prevalence of recent overdose by interest in treatment with injectable diacetylmorphine, a significantly greater proportion of participants interested in treatment with injectable diacetylmorphine reported having ever overdosed (59% vs. 42%).

In adjusted analyses (Table 2), factors significantly associated with interest in treatment with injectable diacetylmorphine included: having at least a high school education (adjusted prevalence ratio [aPR]: 1.23; 95% confidence interval [CI]: 1.04–1.45), not having health insurance (aPR: 1.23; 95% CI: 1.06–1.44), having ever overdosed (aPR: 1.20; 95% CI: 1.01–1.42), and past MOUD-based drug treatment engagement (aPR: 1.22; 95% CI: 1.01–1.47). We also found that recent non-injection cocaine use was inversely associated with interest in treatment with injectable diacetylmorphine (aPR 0.80; 95% CI: 0.68-0.94).

**Table 2.** Poisson regression with robust variance for interest in treatment with injectable diacetylmorphine among PROMOTE participants; Baltimore, MD, April–July 2019.

	Unadjusted			Adjusted		
Variable	PR	95% CI	*p*-Value	aPR	95% CI	*p*-Value
DEMOGRAPHICS						
Age	0.99	0.99 − 1.00	**0.021**	1.00	0.99 − 1.00	0.419
Race/Ethnicity (reference: non-Hispanic, White)						
Non-Hispanic, Black	0.79	0.66 − 0.95	**0.014**	0.86	0.69 − 1.07	0.179
Hispanic or other	0.99	0.78 − 1.25	0.912	0.92	0.71 − 1.21	0.560
STRUCTURAL VULNERABILITIES						
High school diploma/GED or more education	1.21	1.01 − 1.45	**0.034**	1.23	1.04 − 1.45	**0.015**
Food insecurity	1.22	1.02 − 1.46	**0.026**	1.12	0.94 − 1.33	0.206
No health insurance	1.33	1.14 − 1.54	**<0.001**	1.23	1.06 − 1.44	**0.008**
SUBSTANCE USE						
Use drugs in public location, past 3 months	1.27	1.05 − 1.55	**0.016**	1.10	0.90 − 1.34	0.348
Non-injection crack/cocaine^a^	0.85	0.72 − 0.99	**0.038**	0.80	0.68 − 0.94	**0.006**
Injection drug use^a^	1.23	1.06 − 1.44	**0.008**	1.13	0.97 − 1.33	0.127
Ever overdose	1.23	1.04 − 1.45	**0.013**	1.20	1.01 − 1.42	**0.038**
DRUG TREATMENT						
MOUD Status (reference: never MOUD)						
Past MOUD	1.23	1.02– 1.48	**0.029**	1.22	1.01 − 1.47	**0.037**
Currently receiving MOUD	0.86	0.67 − 1.12	0.265	0.94	0.74 − 1.20	0.614

^a^At least monthly.

PR: prevalence ratio.

aPR: adjusted prevalence ratio.

Bold values signify *p*<.05.

## Discussion

To the best of our knowledge, this is the first study to examine interest in treatment with injectable diacetylmorphine among a sample of PWUO in the US. Overall, we found that most PWUO in our sample reported interest in treatment with injectable diacetylmorphine. Factors associated with increased interest in treatment with injectable diacetylmorphine included having at least a high school education, having ever overdosed, and not having health insurance. We also found that past utilization of MOUD-based treatment was associated with interest in treatment with injectable diacetylmorphine (compared to persons who reported having never used MOUD). Persons who reported non-injection crack/cocaine use had lower odds of interest in treatment with injectable diacetylmorphine. Taken together, this study suggests that treatment with injectable diacetylmorphine may be a form of substance use treatment that holds great promise for PWUO in the US, particularly persons who have previously utilized MOUD, but subsequently resumed substance use.

Many participants in our study indicated using multiple classes of substances. It is plausible that persons who use stimulants in combination with opioids may perceive treatment with injectable diacetylmorphine as an incomplete form of treatment for the totality of their substance use. This finding may partially explain our finding that non-injection crack/cocaine use was associated with lower odds of interest in treatment with injectable diacetylmorphine. Research suggests that individuals who use multiple classes of drugs are less likely to receive MOUD [55]. Future research should be conducted to better understand how to comprehensively address treatment needs among persons who have an OUD and concurrently use other substance use classes.

Existing studies have found that persons experiencing a non-fatal overdose is associated with a number of subsequent behavioral changes that may lower risks for future overdose, such as not using drugs alone, testing drugs for fentanyl, and beginning drug treatment [56,57]. In light of these findings, the association we found between persons having ever overdosed and interest in treatment with injectable diacetylmorphine should be explored in future research. Our findings suggest that PWUO and experience an overdose may have increased willingness to engage in treatment with injectable diacetylmorphine if it were available. Further, treatment with injectable diacetylmorphine may prove to be an effective strategy to support recovery for persons who previously accessed existing forms of MOUD available in the US, but resumed substance use and overdosed.

We found that prior utilization of MOUD-based treatment was associated with greater odds of interest in treatment with injectable diacetylmorphine. This finding is most likely explained by a combination of factors. Research has shown that MOUD does not benefit persons equally [26–28]. For example, some persons may not be able to tolerate the side effects. In other instances, persons may not be able to achieve therapeutic dosages of MOUD. Members of this subset of PWUO may perceive treatment with injectable diacetylmorphine as a form of treatment that may overcome the shortcomings of existing forms of MOUD, contributing to their increased interest. Notably, studies have also documented that treatment with diacetylmorphine is more effective than methadone for persons with chronic treatment-refractory heroin dependence [44–46]. This fact, combined with the prevalence of prior MOUD utilization in our sample, underscores the need for making treatment with injectable diacetylmorphine available in the US. Existing efforts to mitigate the addiction and overdose crisis are inadequate and international evidence demonstrates that treatment with diacetylmorphine is another evidence-based strategy for supporting the well-being of PWUO.

This analysis has several limitations that should be considered. Most importantly, treatment with injectable diacetylmorphine is not currently available in the US; as a result, it is plausible that participants had variable interpretations of what treatment with injectable diacetylmorphine entails. Prior to gauging interest, we provided participants with a brief description of treatment with diacetylmorphine. However, our description of treatment with diacetylmorphine only specified ‘legally prescribe injectable heroin…’ rather than other formulations. Future work should be conducted to explore how treatment with diacetylmorphine acceptability may vary across formulations and potential requirements for utilization. Additionally, our framing of injectable diacetylmorphine did not explicitly reference it as a treatment for OUD. Another limitation of this research is that we had limited sample size to explore treatment with injectable diacetylmorphine acceptability across diverse racial and ethnic groups. We also did not provide participants with a definition for ‘overdose’. Given that participants may interpret the term ‘overdose’ differently (e.g. overdosing to the point of passing out versus overdosing and requiring naloxone administration or emergency services), future study should be devoted to exploring how diverse experiences with overdose, including having witnessed an overdose, are associated with interest in treatment with diacetylmorphine. Our data only reflect persons recruited in Baltimore and may not be generalizable across diverse cultures and contexts in the US.

Another limitation is that acceptability for injectable diacetylmorphine may vary based on prior experiences with MOUD and the number and types of substances used. We asked participants about recent MOUD utilization as a single item. As such, we cannot disentangle the relationships between specific MOUD formulations recently used and interest in injectable diacetylmorphine. Studies should be conducted with larger and more geographically diverse samples to holistically assess the relationships between complex profiles of polysubstance use, histories of MOUD utilization, and acceptability for injectable diacetylmorphine. Finally, we are not able to discern with these data the reasons underpinning interest in using injectable diacetylmorphine; it is possible that interest is affected by the route of administration and the medication itself. These limitations notwithstanding, our study fills an important gap in the scientific literature by examining factors associated with interest in a form of drug treatment that is not currently available in the US but is evidence-based and available in other countries.

In conclusion, we found that more than two-thirds of PWUO in our sample reported interest in treatment with injectable diacetylmorphine. Treatment with diacetylmorphine is well-studied internationally with documented benefits for persons with OUD. Expanding access to this evidence-based form of drug treatment in the US requires both additional study and policy change. Given worsening trends in the addiction and overdose crisis through the country, treatment with injectable diacetylmorphine should be considered as another evidence-based option for treating OUD.

## Data Availability

Anonymized data are available upon reasonable request.
